# Mapping of both column acetabular fractures with three-dimensional computed tomography and implications on surgical management

**DOI:** 10.1186/s12891-019-2622-0

**Published:** 2019-05-28

**Authors:** Yun Yang, Chang Zou, Yue Fang

**Affiliations:** Department of Orthopaedics, West China Hospital, Sichuan University, Chengdu, Sichuan People’s Republic of China

**Keywords:** Acetabulum, Fracture, Both-column, Mapping, Computed tomography

## Abstract

**Background:**

The primary goal of this study was to create a frequency map of a series of surgically treated both-column fractures and to explore its implications on surgical management.

**Methods:**

We used a consecutive series of 71 both-column fractures to create 3-dimensional reconstruction images, which were superimposed and oriented to fit a model hemipelvis template by aligning specific pelvis landmarks. Fracture lines were identified and traced to create a fracture map of both-column fractures. Then the possible clinical implications of fracture line map were explored.

**Results:**

Fracture location is closely related to the distribution of fracture line. Of 71 fractures that met the criteria for inclusion, we found the most common pattern demonstrated by coexisting fracture lines. The anterior column was involved by 66% of the fractures extending obliquely from the anterior superior spine to the ischial spine, while 62% of the fractures involved the anterior column extending approximately vertically from the iliac crest to the acetabular roof. Additionally, 39% of the fractures involved the posterior column traversing posterior wall. Furthermore, the high fracture line intensity (*n* = 65, 92%) formed a Y-shaped pattern, which highlighted the consistency of the patterns.

**Conclusions:**

Surgically treated both-column fractures display very common patterns. The most common pattern is the low anterior column fracture in nearly two thirds of cases, the high anterior column fracture in three fifths of cases and the posterior column fracture with posterior wall involvement in nearly two fifths of cases. These study results may help surgeons to yield insight relevant to surgical approaches, reduction, fixation strategies and even implant design for both-column fractures.

## Background

Both-column fractures typically result from high energy trauma, which are complex injuries with technically demanding fracture patterns. In these fractures, the posterior column is usually detached and displaced, possibly with a posterior wall fracture [1]. A secondary fracture line from the posterior column can detach a fragment of the posterior wall that results in the loss of some articular cartilage [2]. If a posterior wall fracture affects the stability of the hip joint in all positions, surgeons often recommend open reduction and internal fixation of the fragment.

In our clinical practice, we have found that associated both-column fractures are frequently associated with posterior wall fractures. However, when posterior wall fractures affect the clinical outcome of both-column fractures, the best way to manage such injuries has not been clearly defined. Many classification systems for acetabular fractures have been established, which may sometimes differ from clinical data sets of actual fracture patterns. A full understanding of the fracture patterns is helpful to develop appropriate surgical strategies. It has been reported that three-dimensional computed tomography (3DCT) reconstruction technology can better understand the three-dimensional structure of acetabular fractures and be used as a guide for surgical planning [3, 4]. The purpose of this study was to analyze 3DCT data from a cohort of patients with a both-column fractures to create a frequency map by superimposing tracked fracture patterns, and to further explore its significance for surgical treatment.

## Methods

### Subjects

At our level I trauma center, a search in the administrative database showed three hundred and seventy two patients with acetabular or pelvic fractures were admitted into the trauma department from January 2009 to January 2018. Inclusion criteria were (1) age of 18 years or older, (2) acute both-column fracture, (3) complete radiographic assessment including X-ray and CT scan. Exclusion criteria included: (1) anatomical details obscured by artifacts, (2) severe comminuted fractures which were difficult to determine fracture lines; (3) poor quality CT data or lack of complete radiographic images. 301 patients were excluded. The remaining 71 patients were analyzed in this study. All radiographs, CT scan and surgical reports were evaluated by two authors to establish the diagnosis.

This study was approved by the Ethics Committee and Institutional Review Board of West China Hospital. All investigations were conducted in conformity with ethical principles of research. The ethics committee waived the need for consent and patients provided verbal consent to participate in the study.

### Fracture mapping

Referring to the mapping method that Cole et al. [5] and Armitage et al. [6] described, we modified the fracture mapping methodology [7] and applied a similar method to the study of both-column fractures. The digitally imaged files (DICOM) were loaded into 3D Slicer (version 4.7.0; Boston, MA, USA), in which three-dimensional computed tomography reconstructions of hemipelvis were created through proper cropping and rotation. Selection of a 3DCT view was based on sufficient evaluation of all images available. These views that allowed the best visualization of the fracture line in the plane (mainly the back of the acetabular posterior column) represented by the standard hemipelvis were collected for each patient. All CT scans for 3D reconstruction had a slide thickness between 0.62 mm and 1.25 mm.

**Fig. 1:**
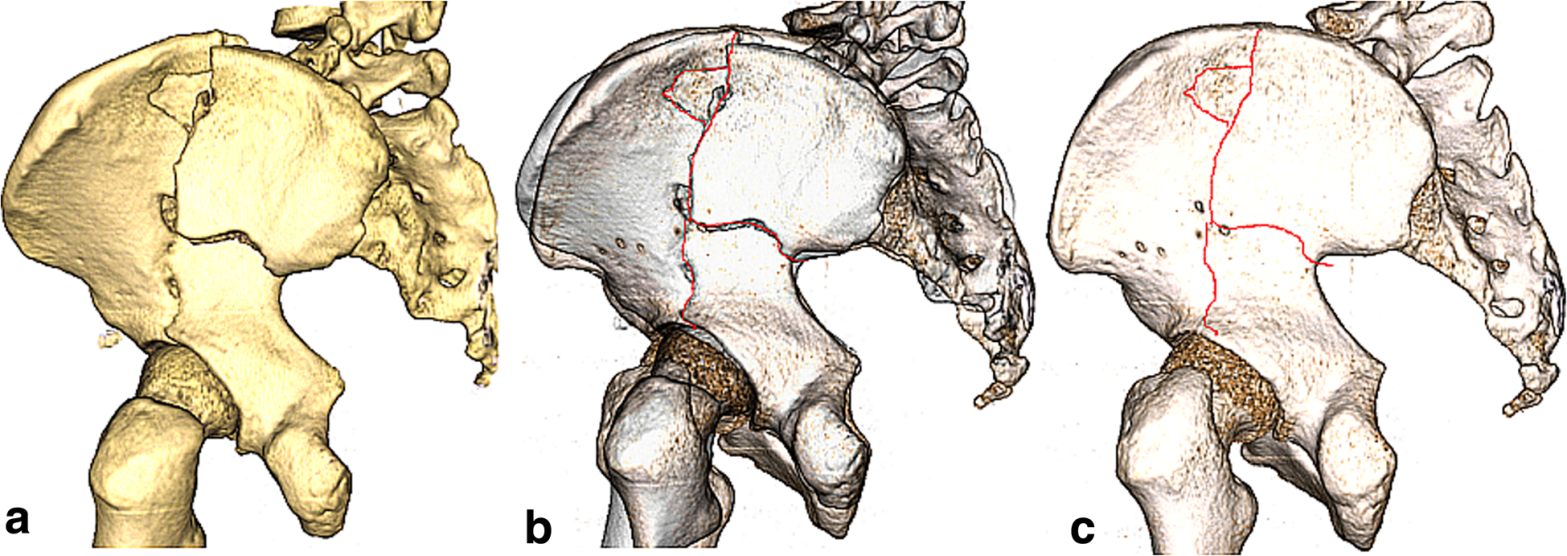
A series of images showing the progression of the mapping of the fractures, starting with (**a**) a computed tomography image, (**b**) fracture line drawn onto the matched standard template, and (**c**) ending with fracture line mapped on the standard template

**Fig. 2:**
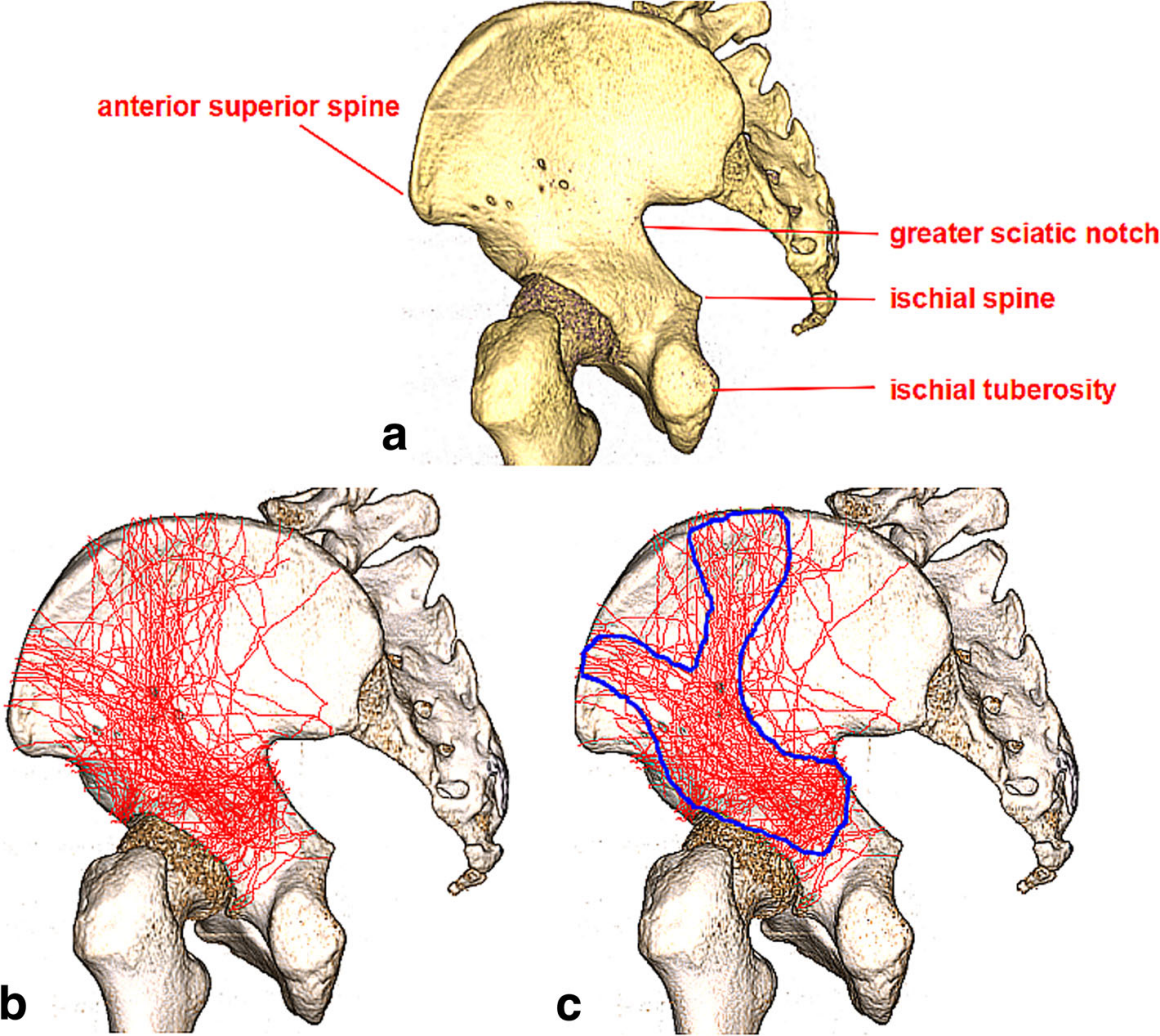
**a** Hemipelvis anatomy. **b** This picture shows the fracture lines of all 71 superimposed fractures. **c** This picture illustrates the “corridors” in which nearly 92 % of the major fracture lines occurred

Then these views were imported into Macromedia Fireworks MX software (Macromedia Inc., San Francisco, CA, USA) to overlap and orient fracture patterns onto a template hemipelvis image. Proper rotation and normalization were guided by aligning specific pelvic landmarks: greater sciatic notch, ischial spine and ischial tuberosity. Once proper anatomical alignment was obtained, fracture lines were identified and were traced on the back of the pelvis (Fig. 1). At the process, lines of each hemipelvis case were graphically superimposed to create a compilation of fracture lines on a standard template of an intact left hemipelvis as a representation of the osseous anatomy. The overlap of all major fracture lines resulted in the creation of a both-column fracture map on the rear surface of the pelvis (Fig. 2b). For back views of right hemipelvis, the images were flipped horizontally to obtain corresponding mirror images. Also, the variability in the size and shape of posterior column was standardized with a 4 × 4 cells grid oriented to the posterior border of the posterior column (greater sciatic notch). All mapping was performed under the guidance of a single resident with a background in computer graphics, and all fracture line interpretations were verified by a trauma fellow and a fellowship-trained trauma surgeon.

In addition, zones were defined on the basis of pelvic anatomy and/or key muscular origins and insertions (Fig. 2a). Based on the definitions of relevant zones, all fractures were analyzed with respect to acetabular involvement. With qualitative and quantitative assessment of fracture map, the direction of the fracture line was further determined.

### Radiographic evaluation and data analysis

Quality of reduction was estimated on anteroposterior (AP) pelvis and Judet views that taken immediately after surgery by Matta’s criteria [8]: anatomical (0–1 mm displacement), imperfect (2–3 mm displacement), and poor (> 3 mm displacement) results. The analysis of the fracture maps was descriptive. Patient characteristics were summarized with frequencies and percentages for categorical variables and with means and standard deviations for continuous variables. The fracture maps were assessed for recurrent patterns of fracture lines and zones of comminution.

## Results

A total of 71 patients met the inclusion criteria, among which were 43 male patients and 28 female patients, with an average age of 41 years. All patients underwent surgery. The most common injury mechanism was fall from height (Table 1). Most both-column fractures (56%) were managed through combined Ilioinguinal and Kocher-Langenbeck (IL + KL) approaches in our study. According to the reduction categories described by Matta, we achieved anatomical reduction in 45 patients (63%), imperfect reduction in fourteen (20%), and poor reduction in twelve (17%). All data were detailed in Tables 2.

**Table 1:** Patient demographics

Variable	All patients (*n* = 71)
Mean age (SD),year	41 (14)
Sex, n (%)	
Male	43 (61)
Female	28 (39)
Side of injury, n (%)	
Right	36 (51)
Left	35 (49)
Injury mechanism, n (%)	
Motor vehicle collision	26 (37)
Fall from height	42 (59)
others	3 (4)
Total	71 (100)

**Table 2:** Surgical approaches and reduction quality

Variable	Results
Surgical approaches, n (%)	
IL	8 (11)
KL	10 (14)
P	3 (4)
S	3 (4)
IL + KL	40 (56)
S + KL	4 (6)
S + IF	2 (3)
S + IF+KL	1 (2)
Reduction status, n (%)	
Anatomical	45 (63)
Imperfect	14 (20)
Poor	12 (17)

*IL* Ilioinguinal, *KL* Kocher-Langenbeck, *P* Pararectus, *S* Stoppa, *IL + KL* Ilioinguinal and Kocher-Langenbeck, *S + KL* Stoppa and Kocher-Langenbeck, *S + IF* Stoppa and Iliac Fossa, *S + IF+KL* Stoppa and Iliac Fossa combined with Kocher-Langenbeck

Consistent fracture lines were identified and deemed major fracture lines by critically reviewing all these images. Clearly, most of major fracture lines entered in three directions as if to replicate a Y-shaped pattern (Fig. 2c). Three corridors, making up the “Y”, can be created to reflect the location of nearly 92 % of all major fracture lines, which further highlighted the consistency of the pattern.

**Fig. 3:**
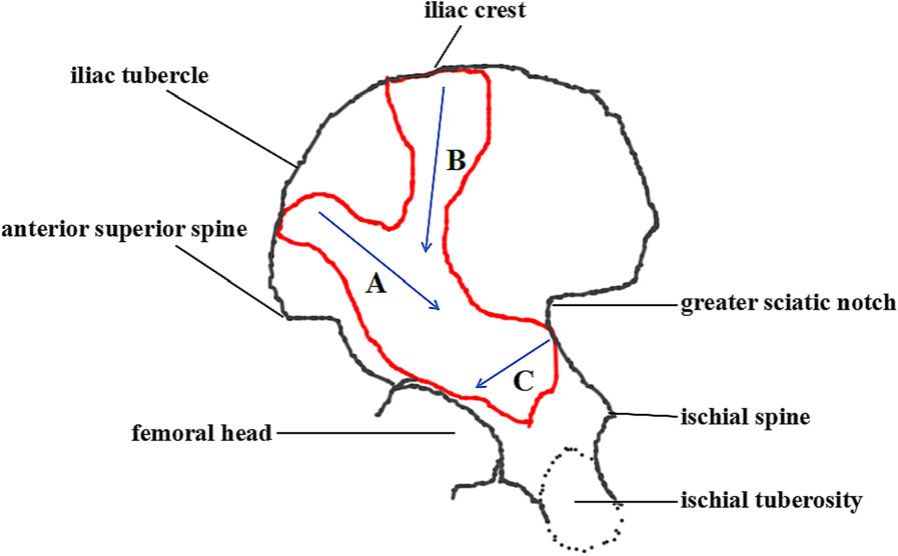
This image shows the area with high incidence of fractures (red line area) and main fracture lines (blue arrow marked with A, B and C)

From Fig. 2b, we were able to find the main fracture lines of both-column fractures. For the convenience of understanding, the fracture line from the point (near the anterior superior spine) between the anterior superior spine and the iliac tuberosity towards the ischial spine was defined as A, that from the iliac crest to the acetabular roof was defined as B, that traversed posterior wall was defined as C (Fig. 3). A and B constituted the main fracture lines of the anterior column in the both-column fractures, while C constituted the main fracture line of the posterior column. Apparently, A can be regarded as low anterior column fracture, while B can be regarded as high anterior column fracture. At the same time, we also found a part of the fractures from the lower part of the anterior superior spine into the anterior column, which can also be considered as low anterior column fractures (Fig. 2b).

**Fig. 4:**
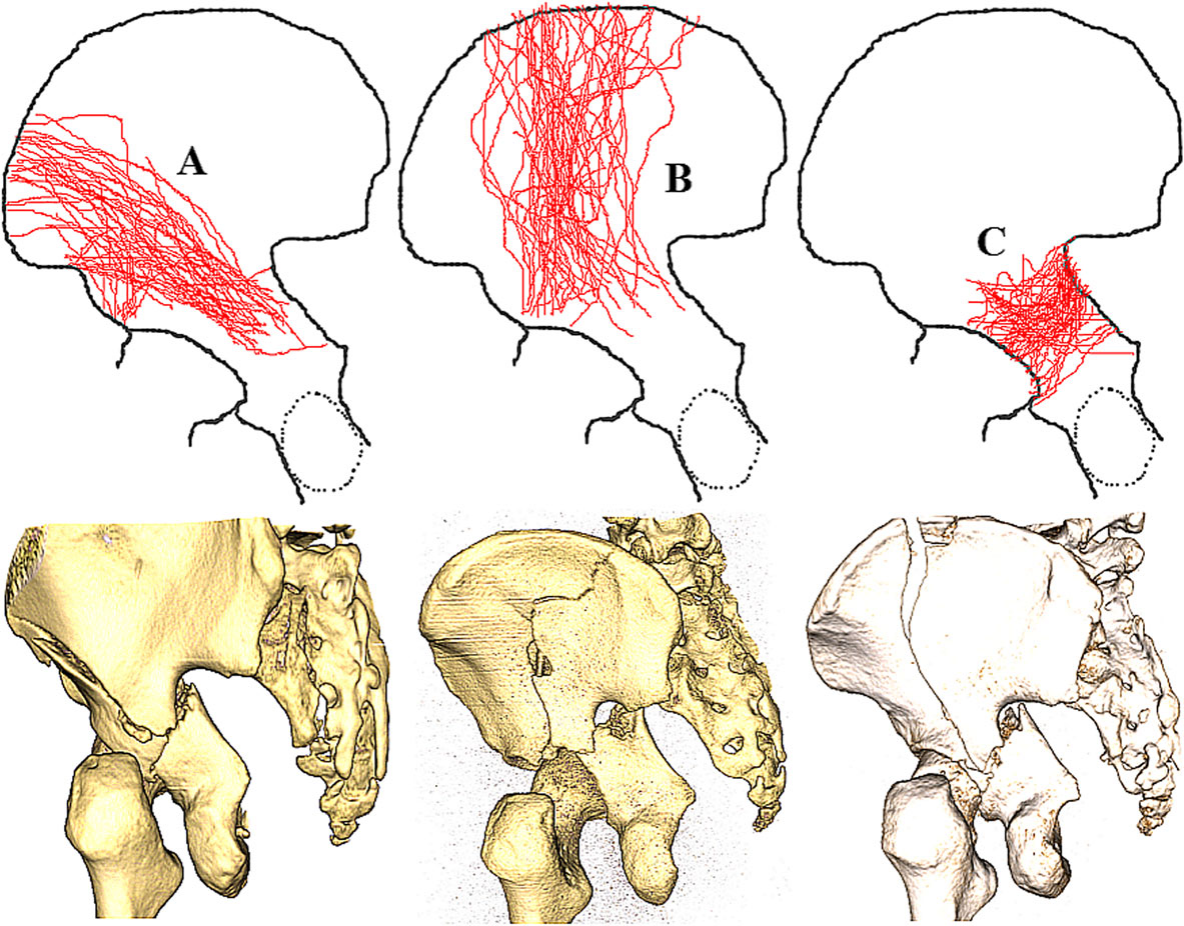
Maps of three major fracture lines marked with **a**, **b** and **c**. Representative three-dimensional computed tomography images of each group are presented for illustrative purposes

According to the above definition, we found that of seventy one both-column fractures, forty four cases (62%) had high anterior column fractures, 47 cases (66%) had low anterior column fractures, twenty-eight cases (39%) had posterior column fractures involving the posterior wall (Fig. 4), and forty-two cases (59%) had posterior column fractures without posterior wall involvement. One case had posterior column fracture involving the sacroiliac joint. In addition, it should be noted that multiple major fracture lines can occur in the same case.

## Discussion

Both-column fractures, characterised by lines developing on multiple planes, are the most complex of all acetabular fractures [9–13]. The detachment of the entire weight-bearing articular surface from the sacroiliac joint is truly pathognomonic of this injury [9–11]. This injury is often caused by lateral direct impact, which is accompanied by central dislocation of the femoral head. In the treatment of such fracture, the specific injury of the acetabular wall, especially the posterior wall, must be defined, as it can affect the surgical approach and prognosis. In addition, we should also consider secondary congruence that might have impact on the indication for surgery. This state would allow hip joint contact stresses to be evenly distributed throughout the articular surfaces. The long-term results after nonoperative management of both-column fractures is better than other fracture patterns that affect the weight-bearing surface of the acetabulum [14–16].

There are various classification schemes for acetabular fractures [14, 17–22], but the Judet-Letournel classification system remains the most widely accepted [14, 17]. The both-column fracture maps can also be helpful to better understand the existing classification system. The OTA/AO classification system [18] divides acetabular fractures into three categories (group A, group B and group C), each of which includes three subcategories (1, 2, and 3). Pierannunzii et al. [23] classified both-column fractures into two subcategories (type I and II) based on the fracture line morphology (T- or Y-sharped). In our study, the both-column fractures of fracture line A and C were consistent with C2 and type I, those of fracture line B and C were consistent with C1 and type II, and those of the posterior column fractures involving the sacroiliac joints were consistent with C3. In addition, the fracture maps in this study revealed common fracture patterns in the anterior and posterior columns of both-column. The incidence of low anterior column fracture was slightly higher than that of high anterior column fracture.

**Fig. 5:**
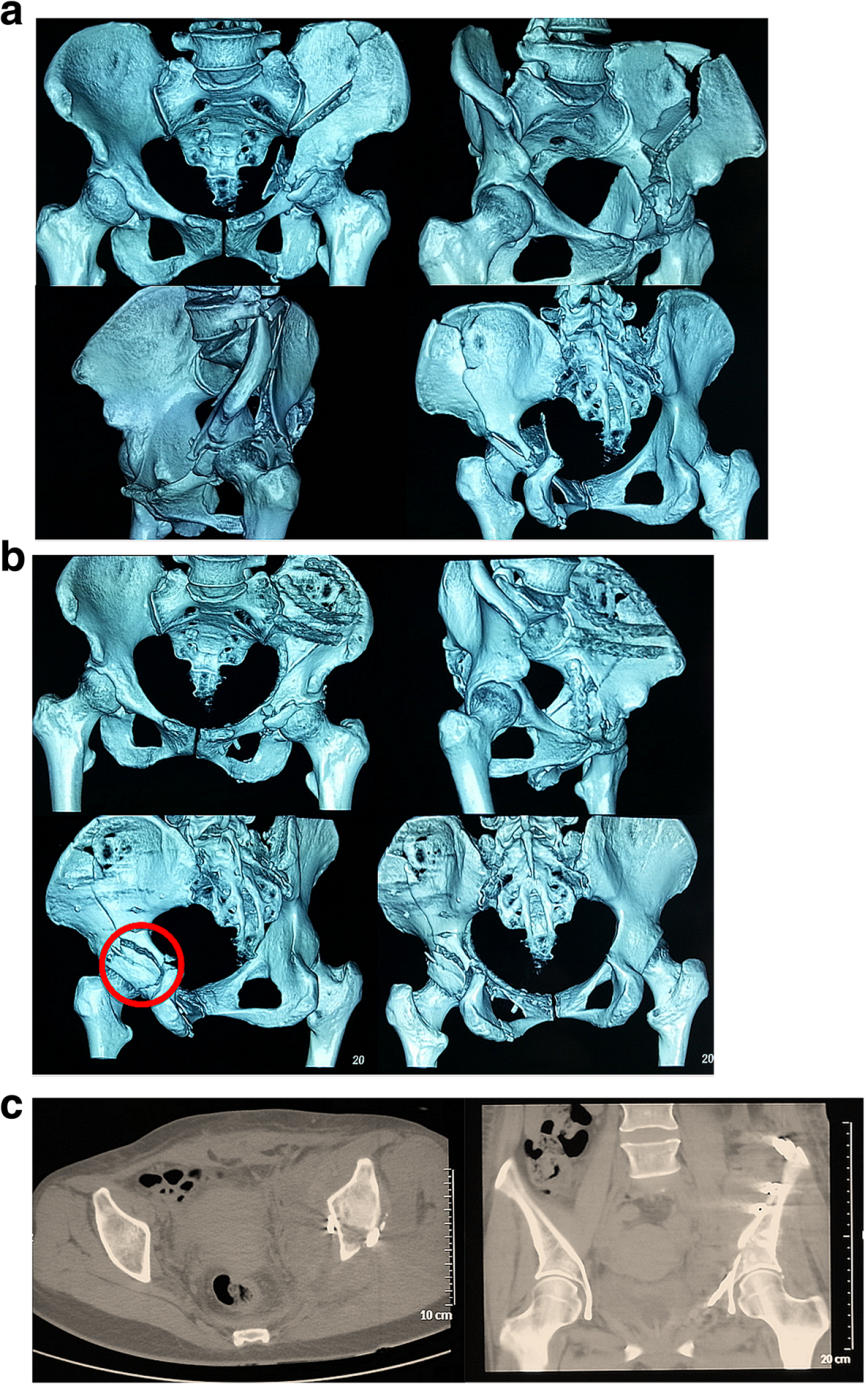
Images for a 45-year-old patient due to a traffic accident. **a** Preoperative computed tomography scans showed a both-column acetabular fracture including high anterior column fracture (fracture line B in the study) and posterior column fracture (fracture line C in the study). **b** high anterior column fracture (fracture line B in the study) was treated through the Stoppa and iliac fossa approach and sequential Kocher-Langenbeck approach was added because of the displaced posterior-wall fragment (red circle). **c** Postoperative coronal and axial CT scans at the level of the acetabular dome presenting anatomic reduction

The choice of surgical approach depends on a variety of factors that include the type of fracture, the direction of fracture displacement, whether accompanied by posterior wall fracture, the conditions of soft tissue and surgeon’s individual preference. The Ilioinguinal approach has been found to be effective in the management of both-column fractures. The specific procedure is that the anterior column fracture is directly reduced through this approach and the posterior column fracture is indirectly reduced, but the premise is that the posterior column fracture is only an isolated large fragment [2, 15]. A single conventional anterior approach is not a good way to deal with posterior wall fractures when double column fractures are combined with posterior wall fractures. Surgical indications for fixation of posterior wall fractures include hip instability, marginal impaction of the articular surface, and intra-articular fragments [24]. Because of the involvement of the posterior wall, some authors recommend the simultaneous addition of Kocher-Langenbeck approach or two-staged procedures for both-column fractures [25, 26]. However, extensile approaches have been reported to be associated with higher rates of complications [25–28]. To optimize treatment and reduce complications, it has been necessary to use limited exposures and understand the common fracture patterns in both-column fractures. Our understanding is that low anterior column fracture (fracture line A in the study) can be reduced and fixed through Stoppa approach, while high anterior column fracture (fracture line B in the study) can be treated through Ilioinguinal approach or combined Stoppa and iliac fossa approach (Fig. 5).

In this study, we identified a Y-shape region with a high incidence of comminution composed of three major fracture lines; these included (1) the fracture line from the point near the anterior superior spine towards the ischial spine, (2) that from the iliac crest to the acetabular roof, and (3) that traversed posterior wall. Furthermore, in the cases of both-column fractures we studied, posterior wall fractures tended to be concomitant. The most common pattern was a fracture line traversing posterior wall.

These results can facilitate the identification of critical locations at which to access displaced fractures. Surgeons can apply our study results to guide the placement of implants for optimal screw purchase and internal fixation. Additionally, we can also combine anatomical parameters of the pelvis with the main fracture lines to design a novel internal fixation device, which is the focus of further research in the future.

Our study has several limitations. First, it was a retrospective review of prospectively gathered data for a small number of patients. Second, our analysis did not take potential variability in anatomy and injury mechanism into account. Third, some hemipelvis images did not match the hemipelvis model perfectly. Finally, fracture lines drawn on the hemipelvis model could slightly differ from true fracture morphology.

## Conclusions

In conclusion, the fracture maps of both-column fractures derived from 3D CT can aid in guiding the treatment of such complex fractures, in terms of surgical approaches, preoperative planning, and implant strategy.

## Abbreviations

3DCT: Three-dimensional computed tomography
AP: Anteroposterior
CT: Computed Tomography
DICOM: Digital Imaging and Communications in Medicine
IL: Ilioinguinal
KL: Kocher-Langenbeck
